# Inflammation, Coagulation and Cardiovascular Disease in HIV-Infected Individuals

**DOI:** 10.1371/journal.pone.0044454

**Published:** 2012-09-10

**Authors:** Daniel A. Duprez, Jacqueline Neuhaus, Lewis H. Kuller, Russell Tracy, Waldo Belloso, Stephane De Wit, Fraser Drummond, H. Clifford Lane, Bruno Ledergerber, Jens Lundgren, Daniel Nixon, Nicholas I. Paton, Ronald J. Prineas, James D. Neaton for the INSIGHT SMART Study Group

**Affiliations:** 1 University of Minnesota, Minneapolis, Minnesota, United States of America; 2 University of Pittsburgh, Pittsburgh, Pennsylvania, United States of America; 3 University of Vermont, Burlington, Vermont, United States of America; 4 Hospital Italiano de Buenos Aires, Buenos Aires, Argentina; 5 Saint-Pierre Hospital, Brussels, Belgium; 6 Kirby Institute, Faculty of Medicine, University of New South Wales, Sydney, Australia; 7 National Institute of Allergy and Infectious Diseases, National Institutes of Health, Bethesda, Maryland, United States of America; 8 University Hospital Zurich, University of Zurich, Zurich, Switzerland; 9 University of Copenhagen, Copenhagen, Denmark; 10 Virginia Commonwealth University, Richmond, Virginia, United States of America; 11 Medical Research Council Clinical Trials Unit, London, United Kingdom; 12 Wake Forest University School of Medicine, Winston-Salem, North Carolina, United States of America; UCL Institute of Child Health, University College London, United Kingdom

## Abstract

**Background:**

The SMART study was a trial of intermittent use of antiretroviral therapy (ART) (drug conservation [DC]) versus continuous use of ART (viral suppression [VS]) as a strategy to reduce toxicities, including cardiovascular disease (CVD) risk. We studied the predictive value of high sensitivity C-reactive protein (hsCRP), interleukin-6 (IL-6) and D-dimer with CVD morbidity and mortality in HIV-infected patients who were enrolled in SMART beyond other measured CVD risk factors.

**Methods:**

A blood sample was available in 5098 participants who were enrolled in the SMART study for the measurement of IL-6, hsCRP and D-dimer. Hazard ratios (HR) with 95% CI for CVD events were estimated for each quartile (Q) for each biomarker vs the 1^st^ quartile and for 1 SD higher levels. For both treatment groups combined, unadjusted and adjusted HRs were determined using Cox regression models.

**Results:**

There were 252 participants who had a CVD event over a median follow-up of 29 months. Adjusted HRs (95% CI) for CVD for Q4 vs Q1 were 4.65 (2.61, 8.29), 2.10 (1.40, 3.16), and 2.14 (1.38, 3.33) for IL-6, hsCRP and D-dimer, respectively. Associations were similar for the DC and VS treatment groups (interaction p-values were >0.30). The addition of the three biomarkers to a model that included baseline covariates significantly improved model fit (p<0.001). Area under the curve (AUC) estimates improved with inclusion of the three biomarkers in a model that included baseline covariates corresponding to other CVD risk factors and HIV factors (0.741 to 0.771; p<0.001 for difference).

**Conclusions:**

In HIV-infected individuals, IL-6, hsCRP and D-dimer are associated with an increased risk of CVD independent of other CVD risk factors. Further research is needed to determine whether these biomarkers can be used to improve CVD risk prediction among HIV positive individuals.

## Introduction

Advances in the management of HIV disease and antiretroviral therapy (ART) have led to a prolonged disease-free survival in a substantial majority of HIV-infected individuals [1], [2]. In parallel with these therapeutic advances, it has become clear that some ART also have serious adverse effects both in the short and long term [3], [4]. Cardiovascular disease (CVD) is now a leading cause of death among HIV-infected individuals and rates of CVD appear to be increased in HIV versus non-HIV infected groups [5]–[7]. Reports from the D:A:D study implicated ART, and protease inhibitor (PI) use in particular, as a possible determinant of CVD risk [8], [9].

Findings from the Strategies for Management of Anti-Retroviral Therapy (SMART) trial fueled interest in the hypothesis that some component of CVD risk may be a consequence of HIV infection itself [10]. The SMART study was designed to examine intermittent use of ART as a strategy to reduce toxicities, including CVD risk. However, despite one-third less antiretroviral drug exposure, intermittent use of ART led to an increased CVD risk compared to continuous use of ART [11].

Studies have shown that HIV replication is an important determinant of endothelial dysfunction [12], [13]. Immune activation and inflammation may explain some of the excess cardiovascular risk associated with HIV [14], [15]. As a consequence of impaired endothelial function and inflammation, HIV-infected individuals may also be in a hypercoagulable state [16]. In previous SMART reports, we showed that baseline levels of IL-6, hsCRP and D-dimer were all strongly related to all-cause mortality [17], and that IL-6 and hsCRP, but not D-dimer, were associated with the development of AIDS events [18]. In this SMART report we examine the association of these biomarkers with CVD morbidity and mortality.

## Methods

### Ethics Statement

Prior to randomization, patients were asked to consent to storing blood for future research. The SMART study, including the consent for stored specimens, was approved by the institutional review board or ethics committee of each clinical site and of the University of Minnesota.

### Design

The design, methods and results of the SMART trial have been previously published [10]. Between January 2002 and January 2006, 5,472 HIV-infected patients with a CD4+ T cell count >350 cells/mm^3^ were randomized to intermittent ART (drug conservation, DC) or continuous ART (viral suppression, VS). For patients in the DC group, ART was not used until the CD4+ count declined to <250 cells/mm^3^, at which time ART was initiated (or reinitiated) until the CD4+ count increased to more than 350 cells/mm^3^. VS patients were to use available ART in an uninterrupted manner with the goal of maximal and continuous suppression of HIV replication. As previously reported on January 11, 2006, enrollment was stopped and participants in the DC group were advised to restart ART. All participants were followed until July 11, 2007 (study closure) [19], resulting in a minimum follow-up of 18 months for each participant and a median follow-up of 29 months.

### Biomarkers and Cardiovascular Outcomes

CVD events occurring through study closure were reviewed by an Endpoint Review Committee using pre-specified criteria blinded to treatment group [20]. The CVD composite outcome used in this report includes: CVD death, non-fatal myocardial infarction (MI) (clinical and silent as measured by annual resting ECG), non-fatal stroke, congestive heart failure (CHF), coronary revascularization, coronary artery disease requiring drug treatment, and peripheral arterial disease. Cause of death was determined using the Coding of Death in HIV (CoDe) system [21]. In this report, 19 deaths of unknown causes that were unwitnessed were considered CVD on the assumption that most would be CVD-related. These unwitnessed deaths do not include violent deaths and deaths attributed to suicide, substance abuse, and accidents by the Endpoint Review Committee. In selected analyses, CVD events are grouped as non-fatal coronary heart disease (CHD) (MI, coronary revascularization, and coronary artery disease requiring drug treatment), non-fatal atherosclerotic non-CHD (stroke and peripheral vascular disease), congestive heart failure (CHF) and CVD death.

Based on strong associations of hsCRP, IL-6 and D-dimer with all-cause mortality in a nested case-control study [17] and the observation that these biomarkers were elevated in HIV-infected participants compared to those in the general population [22], these three markers were measured on stored plasma at baseline for all consenting participants by the Laboratory for Clinical Biochemistry Research at the University of Vermont. IL-6 was measured with Chemiluminescent Sandwich enzyme-linked immunosorbent assay (R&D Systems, Minneapolis, MN); hsCRP with a NM™ II nephelometer, N Antiserum to Human CRP (Siemens Diagnostics, Deerfield, IL); and D-dimer levels with immunoturbidometric methods on the Sta-R analyzer, Liatest D-DI (Diagnostica Stago, Parsippany, NJ). Lipids were centrally measured on serum by Quest Diagnostics, Inc. Lipids were measured on the Olympus AU5400. LDL cholesterol was directly measured. Fasting status influences triglyceride levels but has little effect on total and HDL cholesterol levels; 52% of sample obtained at baseline were fasting. Biomarkers and lipids were analyzed blinded to CVD event status and treatment group.

### Statistical Methods

Time-to-event methods (Cox regression and Kaplan-Meier curves) were used to study associations of baseline levels of each biomarker with the CVD event. Our primary analysis focuses on the DC and VS groups combined. Separate analyses by quartile of each biomarker (defined using the entire cohort) were performed and hazard ratios (HRs) for each of the three upper quartiles versus the lowest quartile (reference group) are cited along with 95% confidence intervals (CIs). In addition to models that categorized the three biomarkers according to quartiles, models with log_10_ transformed biomarkers were considered and the parameter estimates were used to determine the increase in risk of CVD associated with a one standard deviation (SD) higher biomarker level. Adjusted HRs were obtained by considering the following covariates: age, gender, race, ART use, plasma HIV RNA level, CD4+ count, prior AIDS diagnosis, smoking, BMI, prior CVD, diabetes, hypertension treatment, hyperlipidemia treatment, total/HDL cholesterol, presence of major ECG abnormalities [23], hepatitis B or C co-infection and treatment group.

HRs for different types of CVD events and analyses that considered multiple CVD events per patient were also carried out. The consistency of HRs for the different types of events associated with each biomarker were assessed with chi-square [24]. Separate analyses were also considered for the DC and VS groups separately, and results from models that included an interaction term between treatment group and log_10_ biomarker levels are cited.

To determine whether the addition of IL-6, hsCRP, and D-dimer to other CVD risk factors and HIV-related measurements (i.e., the covariates cited above), improved model fit and risk prediction, we carried out a 3 degree of freedom likelihood ratio test and compared area under the receiver operating characteristic curve (AUC) (c index) for the model that included the three biomarkers plus baseline covariates mentioned above and for the model that only included the baseline covariates. The AUC for censored data estimates the probability that, of two randomly chosen patients, that the patient with the higher prognostic score remains free of CVD longer [25]. We used a method for estimating AUC after 29 months (the median follow-up of the cohort) described by Chambless and Diao (method 1 in their report) [26]. Statistical analyses were performed using SAS (Version 9.2, Carey, NC). All reported p-values are 2-sided; p<0.05 is considered statistically significant.

## Results

**Figure 1:**
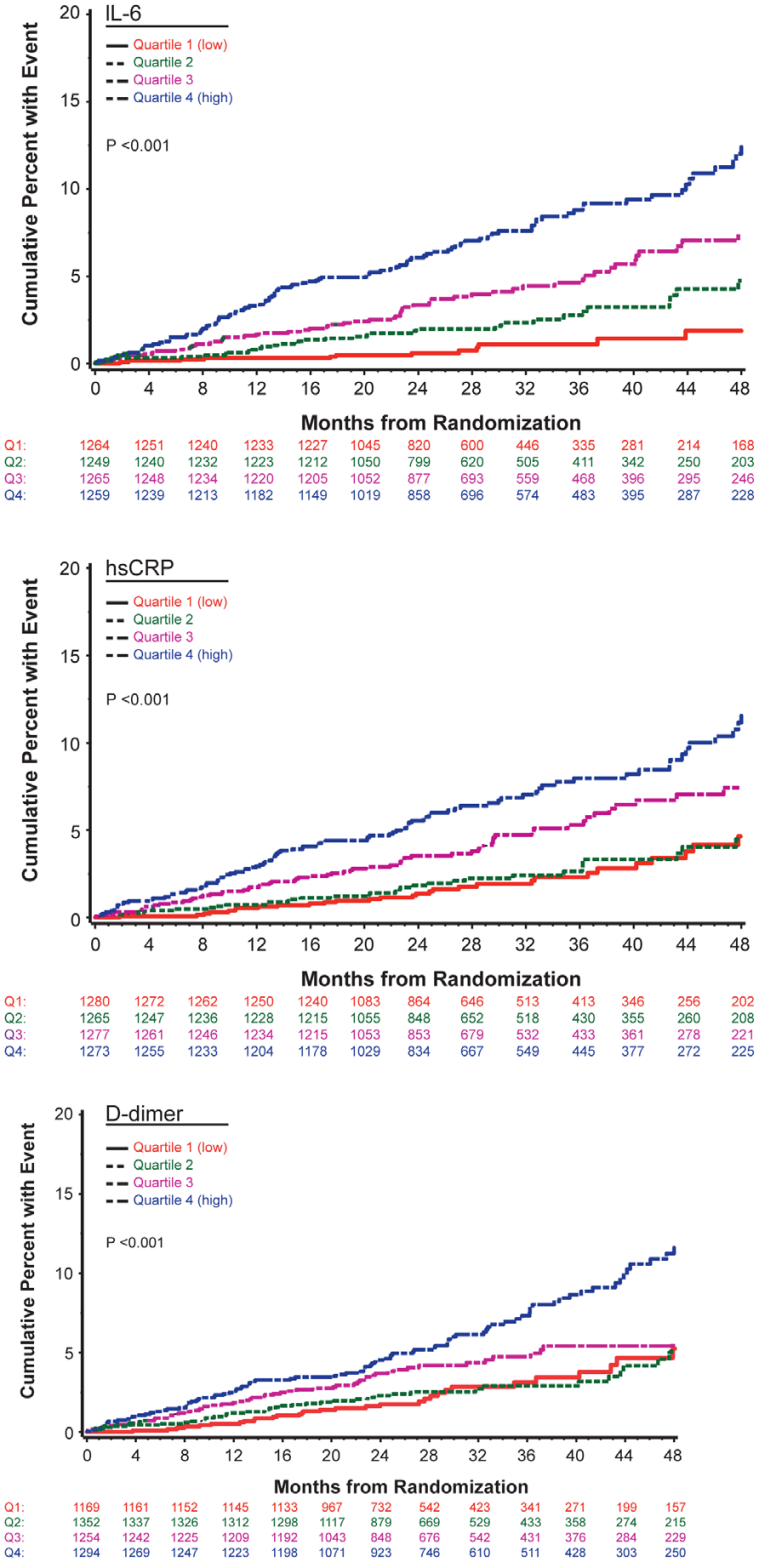
Kaplan-Meier Curves of Cardiovascular Disease (CVD) Events for Interleukin-6 (IL-6), High Sensitivity C-Reactive Protein (hsCRP) and D-dimer. Cumulative percent of participants developing CVD by quartile of IL-6 (percentile cut-off points for IL-6 quartiles are: <1.10, 1.10–1.76, 1.77–3.01, >3.01 pg/mL) (top); cumulative percent of participants developing CVD by quartile of hsCRP (percentile cut-off points for hsCRP quartiles are: <0.72, 0.72–1.71, 1.72–4.17, >4.17 µg/mL) (middle); cumulative percent of participants developing CVD by quartile of D-dimer (percentile cut-off points for D-dimer quartiles are: <0.13, 0.13–0.21, 0.22–0.37, >0.37 µg/mL) (bottom).

During a median follow-up of 29 months, 252 participants experienced at least one CVD event. Numbers experiencing each type of event were: CVD death (n = 44), non-fatal MI (n = 67), non-fatal stroke (n = 20), CHF (n = 30), coronary revascularization (n = 63), coronary artery disease requiring drug treatment (n = 51), and peripheral arterial disease (n = 51). Fifty-four participants experienced more than one CVD event. Table 1 summarizes differences in major CVD risk factors and HIV-related factors for participants with and without CVD events. P-values corresponding to univariate associations and to associations that adjust for age and gender are shown. hsCRP, IL-6 and D-dimer were associated with an increased risk of CVD in both the univariate and age and gender adjusted analyses. Kaplan-Meier curves for quartiles (quartile cut-points are given in figure legend) of each biomarker show good separation of the four curves for IL-6 and for the upper two quartiles versus the lower two quartiles for hsCRP and D-dimer (Figure 1).

**Table 1:** Baseline characteristics: Demographics, HIV factors, CVD risk factors and biomarkers for SMART participants who developed a CVD event and those who did not.

	Participants with CVD event (N = 252)	Participants without CVD event (N = 4846)	p-value^1^	p-value^2^
Treatment group (% DC)	56.3	49.8	0.04	0.04
**Demographics**				
Age (median, IQR)	49 (44, 56)	43 (38, 50)	<0.001	NA
Gender (% female)	19.0	26.3	0.02	NA
Black (%)	39.7	28.7	0.02	0.003
Injection drug use (%)	13.1	9.9	0.36	0.30
CD4+ (cells/mm^3^) (median, IQR)	579 (458, 858)	600 (468, 792)	0.12	0.11
CD4+ nadir (cells/mm^3^) (median, IQR)	236 (120, 350)	252 (157, 360)	0.13	0.44
HIV-RNA ≤400 copies/mL (%)	67.7	71.2	0.77	0.60
Prior AIDS-related illnesses (%)	36.5	23.9	<0.001	<0.001
Hepatitis B (%)	2.0	2.5	0.59	0.64
Hepatitis C (%)	20.2	15.0	0.09	0.13
**CVD Risk Factors**				
Current smoker (%)	52.4	40.9	<0.001	<0.001
Diabetes (%)	17.1	6.6	<0.001	<0.001
Prior CVD (%)	13.1	3.2	<0.001	<0.001
Major ECG abnormality (%)	21.1	8.7	<0.001	<0.001
Blood pressure lowering drugs(%)	44.4	18.0	<0.001	<0.001
Lipid lowering drugs (%)	27.8	15.6	<0.001	0.04
BMI (kg/m^2^) (median, IQR)	25.7 (22.3, 28.9)	25.0 (22.5, 28.1)	0.75	0.34
**Lipids**				
Total cholesterol (mg/dL) (median, IQR)	197 (171, 233)	191 (163, 221)	0.003	0.08
HDL cholesterol (mg/dL) (median, IQR)	38 (31, 49)	41 (33, 51)	0.03	0.04
LDL cholesterol (mg/dL) (median, IQR)	111 (89, 141)	112 (90, 135)	0.27	0.66
Triglycerides (mg/dL) (median, IQR)	192 (134, 308)	163 (105, 260)	0.05	0.30
Total/HDL cholesterol (median, IQR)	5.2 (3.9, 6.8)	4.6 (3.6, 5.9)	<0.001	0.005
**Inflammation and Coagulation Biomarkers**				
hsCRP (µg/mL) (median, IQR)	3.34 (1.47, 7.51)	1.67 (0.70, 4.02)	<0.001	<0.001
IL-6 (pg/mL) (median, IQR)	3.07 (1.87, 4.83)	1.72 (1.07, 2.92)	<0.001	<0.001
D-dimer (µg/mL) (median, IQR)	0.31 (0.18, 0.59)	0.20 (0.13, 0.36)	<0.001	<0.001

1 P-values from univariate Cox models.

2 P-values adjusted for age and gender. Log_10_ transformed levels were used for biomarker analyses.

CVD: Cardiovascular Disease, DC: Drug Conservation, BMI: Body Mass Index, NA: not applicable.

**Figure 2:**
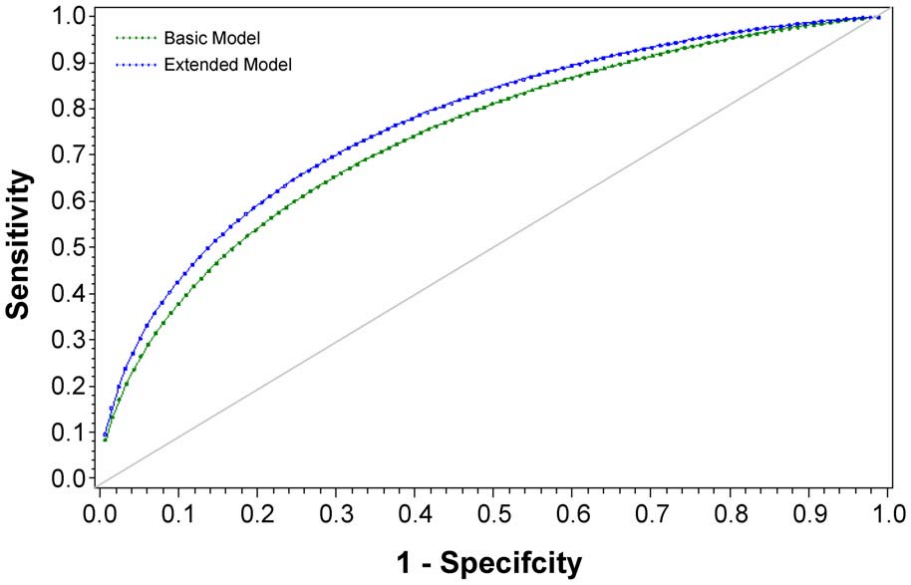
Receiver-Operating Characteristic (ROC) Curve for “Basic” and “Extended” Models for 29 Month Risk of Cardiovascular Disease (CVD) in SMART. The “basic” model included the following baseline covariates: age, gender, race, ART use, plasma HIV RNA level, CD4+ cell count, prior AIDS diagnosis, smoking, BMI, prior CVD, diabetes, hypertension treatment, hyperlipidemia treatment, total/HDL cholesterol, presence of major ECG abnormalities, hepatitis B or C co-infection and treatment group. The “extended” model includes these covariates plus hsCRP, IL-6 and D-dimer after log_10_ transformation.

In a regression model that included all three biomarkers and baseline covariates used for adjustment (see Methods), higher levels of IL-6 (p<0.001), hsCRP (p = 0.003), and D-dimer (p = 0.002), older age (p<0.001), male gender (p = 0.04), higher CD4+ T cell count (p = 0.02), prior AIDS (p = 0.01), smoking (p = 0.002), prior CVD (p = 0.02), diabetes (p = 0.05), antihypertensive therapy (p<0.001), and the presence of major ECG abnormalities (p = 0.03) were associated with an increased risk of CVD. The addition of the three biomarkers to the model that included other baseline covariates significantly improved model fit (likelihood ratio Χ^2^(3df) = 52.2; p<0.001. AUC estimates at 29 months based on models without and with the three biomarkers were 0.741 and 0.771 (p<0.001 for difference). The two ROC curves are shown in Figure 2. The “basic” model refers to the model based on the covariates used for adjustment and cited in Methods and in the figure legend. The “extended” model includes all of the covariates in the “basic” model plus the three biomarkers.

Table 2 gives adjusted HRs of CVD for each biomarker considered separately. Risk gradients with CVD were evident for each biomarker in the quartile analysis and also for the models which treated each biomarker as a continuous variable. For the latter analysis, for each biomarker, a 1 standard deviation (SD) higher level was associated with approximately a 40% increased risk of CVD. As a sensitivity analysis, we repeated these analyses excluding the 19 unwitnessed deaths. The findings were almost identical as those shown in Table 2. For example, adjusted HRs associated with a 1 SD higher level of IL-6, hsCRP, and D-dimer were 1.39 (95% CI: 1.25–1.55), 1.43 (95% CI: 1.23–1.65), and 1.38 (95% CI: 1.19–1.60), respectively.

**Table 2:** Hazard ratios for cardiovascular disease (CVD) associated with baseline biomarker levels.

	IL-6		hsCRP		D-dimer	
BiomarkerQuartile^1^	No. with CVD(Rate per 100person years)	Adjusted HR^2^(95% CI)	No. with CVD(Rate per 100person years)	Adjusted HR^2^(95% CI)	No. with CVD(Rate per 100person years)	Adjusted HR^2^(95% CI)
Lowest	16 (0.5)	1.00	37 (1.1)	1.00	34 (1.1)	1.00
2nd	35 (1.0)	1.68 (0.88–3.18)	36 (1.0)	0.91 (0.57–1.48)	44 (1.2)	1.14 (0.71–1.83)
3rd	67 (1.9)	2.81 (1.55–5.08)	68 (2.0)	1.46 (0.95–2.23)	66 (1.9)	1.57 (1.00–2.46)
Highest	122 (3.5)	4.65 (2.61–8.29)	110 (3.2)	2.10 (1.40–3.16)	105 (2.9)	2.14 (1.38–3.33)
HR^2^ associated with 1 SD^3^ higher (95% CI)		1.39 (1.25–1.54)		1.43 (1.24–1.64)		1.40 (1.21–1.61)

1 Quartile cut-points are given in the legend for Figure 1.

2 Adjusted for age, gender, race, ART, plasma HIV RNA level, CD4+ T cell count, prior AIDS diagnosis, smoking, BMI, prior CVD, diabetes, hypertension treatment, hyperlipidemia treatment, total/HDL cholesterol, ECG abnormalities, hepatitis B or C co-infection, and treatment group.

3 Standard deviations (SDs) after log_10_ transformation were 0.34 pg/mL for IL-6, 0.54 µg/mL for hsCRP, and 0.42 µg/mL for D-dimer.

Associations between IL-6, hsCRP and D-dimer and CVD were similar for the DC and VS groups. Interaction p-values for treatment and log-transformed biomarker levels were 0.39, 0.31, and 0.87 for IL-6, hsCRP, and D-dimer, respectively. Adjusted HRs for 1 SD higher IL-6, hsCRP, and D-dimer were 1.34 (95% CI: 1.17–1.53), 1.57 (95% CI: 1.30–1.89), and 1.35 (95% CI: 1.11–1.64) for DC participants and 1.55 (95% CI: 1.29–1.86), 1.25 (95% CI: 1.01–1.55), and 1.43 (95% CI: 1.16–1.76) for VS participants.

For each biomarker, associations with different types of CVD event did not vary (p = 0.10 for IL-6, p = 0.45 for hsCRP, and p = 0.15 for D-dimer). In an analysis that considered multiple events per patient the adjusted HRs per 1 SD higher level of the biomarker were 1.36 (p<0.001), 1.47 (p<0.001) and 1.43 (p<0.001) for IL-6, hsCRP and D-dimer respectively. Most events were CHD. Adjusted HRs per 1 SD higher level of each biomarker for fatal and non-fatal CHD events or unwitnessed death (n = 160) were 1.37 (1.17, 1.59; p<0.001) for IL-6, 1.18 (1.00, 1.40; p = 0.05) for hsCRP and 1.33 (1.10, 1.61; p = 0.003) for D-dimer. Adjusted HRs comparing the 4^th^ versus 1^st^ quartile for this outcome were 5.23 (p<.001), 1.80 (p = 0.03), and 1.76 (p = 0.06), for IL-6, hsCRP, and D-dimer, respectively.

The joint relationship between IL-6 and D-dimer and between hsCRP and D-dimer with CVD were also considered (Table 3). After log transformation, the correlations between IL-6 and D-dimer and between hsCRP and D-dimer were 0.33 (p<0.001) and 0.20 (p<0.001), respectively. The correlation between IL-6 and hsCRP was 0.47 (p<0.001). When both IL-6 and D-dimer are above the median level the adjusted HR versus those with both IL-6 and D-dimer below the median was 3.96 (95% CI: 2.41–6.51). The p-value for interaction between IL-6 and D-dimer was 0.06. When both hsCRP and D-dimer were considered their effects on CVD risk were additive (p = 0.72 for interaction). When either of hsCRP or D-dimer was above the median risk was increased about 2-fold compared to those with both markers below the median. When both hsCRP and D-dimer were above the median level the adjusted HR versus those with levels below the median was 3.28 (95% CI: 2.04–5.28).

**Table 3:** Joint relationship between IL-6, D-dimer and hs-CRP, D-dimer with cardiovascular disease (CVD).

Bivariate Association of IL-6 and D-dimer with CVD						
IL-6^1^	D-dimer^1^	No. with CVD (Rate per 100 person years)	Unadjusted HR(95% CI)	p-value	Adjusted HR^2^(95% CI)	p-value^2^
<median	<median	26 (0.6)	1.0			
>median	<median	48 (1.9)	2.96 (1.84–4.77)	<.001	2.68 (1.58–4.56)	<.001
<median	>median	25 (1.0)	1.52 (0.88–2.64)	0.133	1.70 (0.93–3.09)	0.084
>median	>median	141 (3.2)	4.86 (3.20–7.39)	<.001	3.96 (2.41–6.51)	<.001
**Bivariate Association of hsCRP and D-dimer with CVD**						
**hsCRP** 1	**D-dimer** 1	**No. with CVD (Rate per 100 person years)**	**Unadjusted HR** **(95% CI)**	**p-value**	**Adjusted HR** 2 **(95% CI)**	**p-value** 2
<median	<median	25 (0.6)	1.0			
>median	<median	52 (1.9)	2.90 (1.80–4.67)	<.001	2.22 (1.33–3.70)	0.002
<median	>median	46 (1.6)	2.39 (1.47–3.90)	<.001	2.01 (1.19–3.40)	0.009
>median	>median	125 (3.0)	4.65 (3.02–7.14)	<.001	3.28 (2.04–5.28)	<.001

1 Quartile cut-points are given in the legend for Figure 1.

2 Adjusted for age, gender, race, ART, plasma HIV RNA level, CD4+ T cell count, prior AIDS diagnosis, smoking, BMI, prior CVD, diabetes, hypertension treatment, hyperlipidemia treatment, total/HDL cholesterol, ECG abnormalities, hepatitis B or C co-infection, and treatment group.

## Discussion

Elevated baseline levels of IL-6, hsCRP and D-dimer were significantly related to CVD and these associations remained after adjustment for CVD risk factors and when considered jointly. The associations of IL-6, hsCRP and D-dimer with different types of CVD events did not differ. Although hyperlipidemia has clearly been linked to the development of atherosclerosis, the potential role of inflammatory mechanisms in the initiation, progression and rupture of the atheromatous plaque has been appreciated only the last decade [27], [28]. A wide range of infections have been associated with persistent inflammation, which itself has been hypothesized to accelerate atherosclerosis [29]. Untreated HIV infection is characterized by increased levels of pro-inflammatory cytokines such as IL-6 and hsCRP, and increased expression of adhesion molecules, factors identified to be important in the pathogenesis of atherosclerosis [14]. These findings suggest that HIV-associated inflammation and associated thrombosis and fibrinolysis are determinants of CVD risk in individuals infected with HIV [30], [31].

Inflammatory cascades are propagated by proximal mediators such as IL-6, which exerts pro-inflammatory effects including stimulation of the liver to produce positive acute-phase proteins during tissue injury or infection. Initially, epidemiological studies of coronary heart disease (CHD) and inflammation focused on “downstream” acute phase reactants, e.g., fibrinogen and hsCRP. In a meta-analysis of 160,309 participants, after adjustment for other CVD risk factors a 1 SD higher CRP level was associated with a 37% higher risk of CHD death, a 55% higher risk of CVD death and a 43% higher risk of non-CVD death [32]. In SMART, a 1 SD higher hsCRP was associated with an 18% increased risk of fatal or non-fatal CHD and a 57% increased risk of fatal or non-fatal CVD. In a previous report, we found that higher levels of hsCRP were significantly related to all-cause mortality [17].

More recently there have been several prospective studies of IL-6 and CHD. In an overview of studies of IL-6 and CHD, a 1 SD higher level of IL-6 was associated with a 26% increased risk [33]. In SMART, a 1 SD higher level was associated with a 39% increased risk of our CVD composite and a 37% increased risk of fatal or non-fatal CHD. In that overview, the authors noted that due to within-person variability (biologic and laboratory variability) the risk of CHD associated with IL-6 was underestimated. With adjustment for this regression dilution bias, they found an 83% increased risk of CHD associated with a 1 SD higher IL-6. The associations we report in SMART are also likely attenuated.

It has been suggested that modestly elevated circulating D-dimer values reflect minor increases in blood coagulation, thrombin formation, and turnover of cross-linked intravascular fibrin and that these increases may be relevant to CHD. D-dimer values may also reflect inflammatory states. In a meta-analysis of D-dimer and CHD, the adjusted odds ratio (upper versus lowest tertile of D-dimer) was 1.79 (95% CI, 1.36 to 2.36) [34]. In our study, risk ratios for the 4^th^ versus 1^st^ quartile were 1.76 for fatal or non-fatal CHD and 2.14 for our CVD composite. In SMART the risk ratios associated with higher D-dimer levels for CVD were much smaller than previously reported risk ratios for all cause mortality [17]. In this respect our findings are similar to findings in older participants in the Multiethnic Study of Atherosclerosis (MESA) [35]. In that report, 6,391 participants were followed for an average of 4.6 years and 307 CVD events, 207 CHD events and 210 deaths were observed. Age, sex, race and risk-factor adjusted hazard ratios for the the 4^th^ versus 1^st^ quartile of D-dimer were 1.08 (95% CI: 0.75–1.55) for CVD, 1.27 (95% CI: 0.80–2.01) for CHD, and 2.57 (95% CI: 1.54–4.27) for all-cause mortality. There have been few studies examining the association of D-dimer with CVD in HIV-infected participants. In a case-control study of 52 CVD events, Ford et al. found that D-dimer levels but not hsCRP or IL-6 levels were significantly elevated 4 months and 2 years prior to developing the CVD event as compared to matched controls [36].

Even though treatment interruption led to an increase in IL-6, hsCRP and D-dimer and treatment initiation led to a decline in D-dimer [14], [37], associations of these markers at baseline with the development of CVD events were similar for DC and VS patients. We have previously shown that these markers are elevated even in patients taking ART with a suppressed viral load [22]. This, coupled with the fact that DC participants were advised to re-initiate ART midway through the study, likely makes it difficult to detect any differences in baseline associations of these markers with risk of CVD that might have been influenced by changes during follow-up. Further research is needed on serial patterns of these markers with different clinical outcomes.

Even though the likelihood-based method of model fit indicated significant improvement (p<0.001) with the addition of the three biomarkers to a model that included other CVD and HIV risk factors, the improvement in CVD risk discrimination as measured by the AUC was modest. Further research on the utility of these biomarkers for classifying participants by CVD risk is needed, including risk reclassification methods with the models described here applied to other cohorts.

There are some limitations of this analysis. We studied the association of a single measurement of the inflammatory markers and coagulation factor at the beginning of the study with CVD. Thus, associations reported are likely underestimated. Also, we studied only a limited number of biomarkers from the inflammatory and thrombotic/fibrinolysis pathways. Strengths of this study are that CVD outcomes were pre-specified and adjudicated by an endpoint review committee and adjustment for most major CVD risk factors was possible.

In conclusion, this study demonstrated that thrombosis and inflammation are inextricably intertwined in the biology of atherosclerosis in HIV-infected individuals. The current findings do not, of course, establish causality, but they may have implications for understanding disease mechanisms and for further research strategies [38]. Further studies are warranted to determine if therapies that result in lowering of these biomarkers lead to reductions in risk of CVD.
